# LDPC Codes on Balanced Incomplete Block Designs: Construction, Girth, and Cycle Structure Analysis

**DOI:** 10.3390/e27050476

**Published:** 2025-04-27

**Authors:** Hengzhou Xu, Xiaodong Zhang, Mengmeng Xu, Haipeng Yu, Hai Zhu

**Affiliations:** 1 School of Computer, Henan University of Engineering, Zhengzhou 451191, China; mmxu873@163.com (M.X.); yuhp@haue.edu.cn (H.Y.); zhu_sea@163.com (H.Z.); 2 School of Mathematical Sciences, Shanghai Jiao Tong University, Shanghai 200240, China; xiaodong@sjtu.edu.cn

**Keywords:** LDPC codes, balanced incomplete block designs (BIBDs), Tanner graph, cycle, girth

## Abstract

In this paper, we investigate the cycle structure inherent in the Tanner graphs of low-density parity-check (LDPC) codes constructed from balanced incomplete block designs (BIBDs). We begin by delineating the incidence structure of BIBDs and propose a methodology for constructing LDPC codes based on these designs. By analyzing the incidence relations between points and blocks within a BIBD, we prove that the resulting LDPC codes possess a girth of 6. Subsequently, we provide a detailed analysis of the cycle structure of the constructed LDPC codes and introduce a systematic approach for enumerating their short cycles. Using this method, we determine the exact numbers of cycles of lengths 6 and 8. Simulation results demonstrate that the constructed LDPC codes exhibit excellent performance.

## 1. Introduction

Low-density parity-check (LDPC) codes exhibit remarkable performance within the framework of iterative decoding. Several structural properties of LDPC codes significantly influence their iterative decoding performance, including their minimum distance, row redundancy, and degree distribution of their parity-check matrices, as well as the girth and cycle distribution of their Tanner graphs [1], among others. For an LDPC code, the iterative decoder, which operates based on belief propagation, functions on its Tanner graph. The presence of cycles causes the decoder to operate locally until a solution is found or the termination condition is met. In other words, the decoding solution is derived primarily around cycles, particularly short cycles, which prevents the decoder from achieving globally optimal solutions. Consequently, iterative decoders operating on Tanner graphs with short cycles are unable to identify maximum-likelihood (ML) code words [2]. As a result, cycles, especially short ones, adversely affect the decoding performance of LDPC codes when decoded using iterative algorithms. This makes the study of cycle distribution in LDPC codes a topic of significant interest [3].

Numerous effective algorithms have been developed for the counting of cycles in LDPC codes (or their Tanner graphs). In bipartite graphs, cycles of lengths g,g+2, and g+4 were enumerated in [4], where *g* represents the girth of the graph. However, the computational complexity of these methods increases rapidly with the number of variable nodes. Several alternative approaches have been proposed based on graph properties such as eigenvalues, degree distribution, and spectrum [5,6,7,8]. Additionally, leveraging message-passing algorithms, two competitive methods were presented in [9,10]. Nevertheless, for the methods described in [5,6,7,8,9,10], the lengths of the counted cycles do not exceed 2g, where *g* is the girth of the LDPC code. Furthermore, necessary and sufficient conditions for the existence of cycles in quasi-cyclic LDPC (QC-LDPC) codes have been established in [11,12,13,14], along with corresponding cycle-counting algorithms. Determining the number of cycles in the Tanner graphs of well-designed LDPC codes based on design rules or structural properties remains a topic of significant interest.

Complementary to these, random-like constructions with carefully designed cycle-avoidance rules have also been developed [15,16,17]. A unifying framework emerges through partial geometries, which provides a geometric perspective for the analysis of code structures [18]. This approach has proven particularly valuable, as partial geometry-based LDPC codes demonstrate exceptional iterative decoding performance [19,20]. Notably, balanced incomplete block designs (BIBDs) constitute an important subclass of partial geometries [21], making them particularly attractive for code construction.

The design of LDPC codes with large girth and a minimal number of short cycles has been approached through various mathematical frameworks. Algebraic constructions utilizing finite fields [22], finite geometries [23], Sidon sequences [24], protographs [25], and transversal designs [26] have shown promising results. Additionally, random-like LDPC codes designed under specific rules (e.g., avoiding or eliminating short cycles) have also been proposed [15,16,17]. Connections among algebraically constructed LDPC codes were explored in [27]. Notably, a well-known general framework called partial geometries was introduced, under which many existing LDPC code constructions can be unified [18]. By leveraging the concept of partial geometries, key structural properties of LDPC codes and their parity-check matrices can be analyzed from a geometric perspective. Research has demonstrated that LDPC codes constructed based on partial geometries exhibit excellent performance under iterative decoding [19,20]. Notably, BIBDs constitute an important subclass of partial geometries [21], making them particularly attractive for code construction. Consequently, the use of BIBDs to construct high-performance LDPC codes has become a topic of significant interest in coding theory [28,29,30,31,32,33]. It is worth noting that the construction methods for some BIBDs were detailed in [34].

In this paper, we investigate LDPC codes constructed based on balanced incomplete block designs (BIBDs). By analyzing the cycle structure, we aim to understand how the design parameters of BIBDs, such as block size and the number of points, affect the presence and distribution of short cycles, which are critical factors in determining the performance of LDPC codes under belief propagation decoding. Unlike previous works reported in [28,29,30,31,32,33], we utilize the complete structure of a BIBD to design LDPC codes. Specifically, given a BIBD, the constructed LDPC code exhibits well-defined parameters, such as code length, row weight, and column weight the parity-check matrix. Moreover, we establish the incidence relationship between points and blocks in a BIBD and analyze the cycle structure within the Tanner graphs of the resulting LDPC codes. Based on this analysis, we propose a systematic method for counting cycles and determine the exact number of cycles of lengths 6 and 8 in the Tanner graphs. Finally, we provide numerical simulation results to demonstrate the performance of the constructed LDPC codes based on BIBDs.

The main contributions of this paper are outlined as follows.

- Novel structured construction: We present a systematic framework for constructing LDPC codes using complete BIBDs. The proposed method generates parity-check matrices with guaranteed structural properties that satisfy the row–column (RC) constraints, extending the partial geometry approach established in [18]. The construction method provides a mathematically rigorous yet practically viable coding solution that bridges the gap between theoretical design and communication requirements. This makes it particularly attractive for applications demanding both excellent error correction capability and implementation feasibility.
- Girth guarantees and performance analysis: We provide a rigorous proof that the BIBD-LDPC codes achieve a minimum girth of 6, effectively eliminating detrimental short cycles that impair iterative decoding. The inherent properties of BIBDs, including balanced connectivity and optimized cycle structure, naturally prevent small trapping sets and low-weight code words, thereby enhancing both waterfall-region performance and error-floor characteristics.
- Comprehensive cycle analysis: We develop a systematic methodology for analyzing the cycle structure of the constructed codes. The proposed technique enables precise enumeration of cycles (particularly lengths 6 and 8) in the Tanner graphs, with a generalizable framework that can be extended to count longer cycles (e.g., 10, 12, etc.). This analysis provides valuable insights into the code’s graphical properties and decoding behavior.

## 2. LDPC Codes Constructed from BIBDs and Their Tanner Graphs

### 2.1. LDPC Codes and Tanner Graphs

An LDPC code is defined by the null space of a sparse matrix ($H=[h_{ij}]$), known as a parity-check matrix. If every row of H contains ρ nonzero elements and every column of H contains γ nonzero elements, the resulting LDPC code is referred to as (γ,ρ)-regular. The Tanner graph of an LDPC code is a bipartite graph, where the two types of vertices correspond to the row indices and column indices of H, respectively. An edge connects the row index (*i*) and the column index (*j*) if and only if element $h_{ij}$ of H is nonzero. A cycle in the Tanner graph is a closed path of interconnected edges that starts and ends at the same edge, traversing no other edge more than once. The length of the shortest cycles in the Tanner graph of an LDPC code is known as its girth.

### 2.2. Balanced Incomplete Block Designs (BIBDs)

Let X be a finite set of points and B be a collection of subsets of X, where the elements of B are referred to as blocks. The number of elements in X is denoted by υ, i.e., |X|=υ. Let the number of blocks in B be *m*, and let *K* denote the set of possible sizes for the blocks. Specifically, each block in B contains a number of elements that belong to *K*. This means that the size of any block in B is one of the values in set *K*, i.e., for $B_{j}\subset B,1\leq j\leq m,\left|B_{j}\right|\in K$. A BIBD is an incidence structure defined by pair (X,B), which satisfies the following properties:

(1) Each block in B contains exactly $k_{i}$ points for $k_{i}\in K$.
(2) Every pair of distinct points in X is contained in exactly λ blocks.

This incidence structure (X,B) is denoted by BIBD(K,λ;υ). For K={κ}, BIBD(K,λ;υ) is denoted by BIBD(κ,λ;υ) for short. In combinatorial design theory, BIBD(K,1;υ) is also referred to as the Steiner 2 design. These properties ensure a balanced and structured relationship between the points and blocks, making balanced incomplete block designs (BIBDs) a cornerstone of combinatorial design theory. Their well-defined and symmetric structure is particularly valuable in the construction of LDPC codes, where BIBDs are employed to achieve desirable properties such as large girth and controlled cycle distributions in Tanner graphs. This makes BIBDs a powerful tool for designing LDPC codes with enhanced iterative decoding performance.

### 2.3. LDPC Codes Constructed Based on BIBDs

Consider a BIBD(K,λ;υ), where the number of blocks in B is *m*. The points and blocks in BIBD(K,λ;υ) are represented as $x_{1},x_{2},\ldots ,x_{\upsilon }$ and $B_{1},B_{2},\ldots ,B_{m}$, respectively, that is, $X=\{x_{1},x_{2},\ldots ,x_{\upsilon }\}$ and $B=\{B_{1},B_{2},\ldots ,B_{m}\}$. Algebraically, BIBD(K,λ;υ) can be represented by an incidence matrix ($I=[I_{ij}]$) of size υ×m, whose elements are either 0 or 1. The rows of I correspond to the points of the BIBD, labeled from 1 to υ (or, equivalently, $x_{1},x_{2},\ldots ,x_{\upsilon }$), and the columns correspond to the blocks, labeled from 1 to *m* (or, equivalently, $B_{1},B_{2},\ldots ,B_{m}$). Element $I_{ij}$ equals 1 (i.e., $I_{ij}$ = 1) if and only if the *i*-th point ($x_{i}$) is contained in the *j*-th block ($B_{j}$); otherwise, $I_{ij}=0$. In this paper, the incidence matrix of a BIBD is used as the parity-check matrix, and its null space defines an LDPC code, referred to as the BIBD-LDPC code. This approach leverages the combinatorial properties of BIBDs to construct LDPC codes with desirable structural characteristics.

To facilitate the understanding of BIBDs, an example is provided. Consider a BIBD(3,1;7). Here, the number of elements in each block is 3, and the cardinality of point set X is 7. Without loss of generality, let the point set be X={a,b,c,d,e,f,g} and the block set be B={{a,e,g},{a,b,f},{b,c,g},{a,c,d},{b,d,e},{c,e,f},{d,f,g}}. A straightforward counting argument confirms that the incidence structure (X,B) satisfies the properties of a BIBD(3,1;7). Consequently, the incidence matrix ($I=[I_{ij}]$) of size 7×7 is constructed as follows:

$$I={\left[I_{ij}\right]}_{7\times 7}=\left[\begin{array}{ccccccc} 1 & 1 & 0 & 1 & 0 & 0 & 0\\ 0 & 1 & 1 & 0 & 1 & 0 & 0\\ 0 & 0 & 1 & 1 & 0 & 1 & 0\\ 0 & 0 & 0 & 1 & 1 & 0 & 1\\ 1 & 0 & 0 & 0 & 1 & 1 & 0\\ 0 & 1 & 0 & 0 & 0 & 1 & 1\\ 1 & 0 & 1 & 0 & 0 & 0 & 1 \end{array}\right],$$

where each row corresponds to a point in X and each column corresponds to a block in B. Element $I_{ij}$ is 1 if the *i*-th point is included in the *j*-th block; otherwise, it is 0. It can be observed that the rank of the incidence matrix (I) is 4. Consequently, the null space of I defines a (3,3)-regular (7,3) LDPC code. This code is characterized by a code length of 7, a dimension of 3, and a parity-check matrix with uniform row and column weights of 3.

To avoid generating cycles of length 4, a well-known row–column (RC) constraint can be applied to the parity-check matrix. Specifically, no two rows (or two columns) in the parity-check matrix should have more than one position in common. Therefore, for BIBD-LDPC codes to be free of cycles of length 4, it is necessary to ensure that any two points do not appear together in more than one block or, equivalently, any two blocks share, at most, one common point. This condition requires that the λ parameter equal 1. Additionally, the column weights of the parity-check matrix take values from set *K*. To ensure that the BIBD-LDPC code has a fixed column weight, we set K={κ}. Consequently, in the remainder of this paper, the discussed BIBD-LDPC codes are constructed from BIBD(κ,1;υ).

According to the structural properties of BIBD(κ,1;υ), the code length of the constructed BIBD-LDPC code is given by

$$\frac{\upsilon \left(\upsilon -1\right)}{\kappa \left(\kappa -1\right)}.$$

The incidence matrix of BIBD(κ,1;υ) has a column weight of κ and a row weight of $\left(\upsilon -1\right)/\left(\kappa -1\right)$. The rank of the incidence matrix depends on the specific structure of the BIBD. Numerical results show that the parity-check matrices of BIBD-LDPC codes typically have full (or nearly full) rank. Therefore, the code rate (*R*) of a BIBD-LDPC code constructed from BIBD(κ,1;υ) is at least

$$R\geq 1-\frac{\kappa \left(\kappa -1\right)}{\upsilon \left(\upsilon -1\right)}.$$

## 3. Girth and Cycle Structure Analysis of BIBD-LDPC Codes

We now consider a BIBD-LDPC code whose parity-check matrix is the incidence matrix (I) of a given BIBD(κ,1;υ). In this section, we analyze the cycle structure of BIBD-LDPC codes, determine their girth, and present a method for counting cycles in their Tanner graphs. Specifically, We investigate how the combinatorial properties of BIBDs influence the cycle distribution in the Tanner graphs of these codes, potentially providing valuable insights into their iterative decoding performance.

### 3.1. Cycle Structure of BIBD-LDPC Codes

According to the definition of cycles in the Tanner graph [1,11], a cycle in the BIBD-LDPC code can be represented as an ordered sequence of positions of alternate nonzero elements in the parity-check matrix. Based on the structure of the incidence matrix of BIBD(κ,1;υ), a cycle of length 2i in the Tanner graph of the BIBD-LDPC code can be expressed as the following sequence:

(1) $$\begin{array}{c} x_{j_{0}},B_{l_{0}},x_{j_{1}},B_{l_{1}},x_{j_{2}},B_{l_{2}},\ldots ,x_{j_{i-1}},B_{l_{i-1}},x_{j_{0}},B_{l_{0}} \end{array}$$

where for $0\leq k\leq i-1,j_{k}\neq j_{k+1},j_{i}=j_{0},l_{k}\neq l_{k+1},l_{i}=l_{0},x_{j_{k}}\in X,$ and $B_{j_{k}}\subset B$. $x_{j_{0}},x_{j_{1}},\ldots ,x_{j_{i-1}}$ are distinct points in X, and $B_{l_{0}},B_{l_{1}},\ldots ,B_{l_{i-1}}$ are distinct blocks in B. Each consecutive pair ($\left(x_{j_{i}},B_{l_{i}}\right)$ and $\left(x_{j_{i+1}},B_{l_{i}}\right)$, where $j_{i}$ = $j_{0}$ and $l_{i}$ = $l_{0}$) corresponds to a nonzero entry in the incidence matrix (I), ensuring the cycle is closed. This sequence represents a closed path in the Tanner graph, where each point ($x_{j_{i}}$) is connected to block $B_{l_{i}}$ and each block ($B_{l_{i}}$) is connected to the next point ($x_{j_{i+1}}$), forming a cycle of length 2i.

In other words, the adjacent points (or blocks) in the sequence (1) are distinct from one another. Additionally, each block in (1) contains its two adjacent points, and each point in (1) is included in its two adjacent blocks. For a BIBD(κ,1;υ), any pair of points in X is contained in exactly one block. Consequently, for two points adjacent to the same block in (1), no other block contains both of them. Therefore, the point-and-block sequence (1) can be simplified and represented by the following point sequence:

(2) $$\begin{array}{c} x_{j_{0}},x_{j_{1}},x_{j_{2}},\ldots ,x_{j_{i-1}} \end{array}$$

where for $0\leq k\leq i-1,j_{k}\neq j_{k+1}$, that is, a cycle of length 2i can be fully represented by the point sequence (2), making it unnecessary to explicitly consider the blocks in the cycle. Therefore, a cycle of length 2i corresponds uniquely to an ordered point sequence of the following form:

$$x_{j_{0}},x_{j_{1}},x_{j_{2}},\ldots ,x_{j_{i-1}},$$

where $x_{j_{0}},x_{j_{1}},x_{j_{2}},\ldots ,x_{j_{i-1}}$ are distinct points in X and each consecutive pair ($x_{j},x_{j+1}$, where $x_{i}=x_{0}$) is contained in exactly one block ($B_{k}\in B$). This representation simplifies the analysis of cycles by focusing solely on the point sequence, leveraging the property of BIBD(κ,1;υ) according to which each pair of points appears in exactly one block. As a result, the cycle structure is entirely determined by the arrangement of points, eliminating the need to explicitly track the blocks in the cycle.

### 3.2. Girth of BIBD-LDPC Codes

Based on the above analysis, we observe that BIBD-LDPC codes are constructed from the BIBD(κ,1;υ) incidence structure, which is (X,B), that is, λ=1. Hence, the parity-check matrices of these BIBD-LDPC codes satisfy the row–column (RC) constraint. This ensures that the constructed BIBD-LDPC codes are free of cycles of length 4 and, consequently, have a girth of at least 6. In fact, the girth of these BIBD-LDPC codes is exactly 6. To further determine the girth of BIBD-LDPC codes, we aim to identify a cycle of length 6 in the Tanner graph of a BIBD-LDPC code, and such a cycle corresponds to the point-and-block sequence described in (1). This sequence represents a closed path of length 6 in the Tanner graph, confirming the presence of cycles of length 6 in the BIBD-LDPC code.

Consider two distinct points ($x_{1},x_{2}\in X$). According to the definition of BIBD(κ,1;υ), there exists a unique block ($B_{1,2}\subset B$) that contains both $x_{1}$ and $x_{2}$. Since $B_{1,2}$ is a proper subset of X, there exists a point ($x_{3}\in X$) such that $x_{3}\notin B_{1,2}$. Consequently, $x_{3}$ is distinct from $x_{1}$ and $x_{2}$. According to the properties of BIBD(κ,1;υ), there exists a unique block ($B_{2,3}\subset B$) that contains both $x_{2}$ and $x_{3}$. It is clear that $B_{1,2}$ and $B_{2,3}$ are distinct blocks because $x_{3}\notin B_{1,2}$ but $x_{3}\in B_{2,3}$. Similarly, based on points $x_{1}$ and $x_{3}$, there exists a unique block ($B_{1,3}\subset B$) that contains both $x_{1}$ and $x_{3}$. Since $x_{2}\notin B_{1,3}$ (while $x_{2}\in B_{1,2}$) and $x_{1}\in B_{1,3}$ (while $x_{1}\notin B_{2,3}$), $B_{1,3}$ is distinct from both $B_{1,2}$ and $B_{2,3}$. Thus, blocks $B_{1,2},B_{2,3}$, and $B_{1,3}$ are all distinct. This leads to the following point-and-block sequence:

$$\begin{array}{c} x_{1},B_{1,2},x_{2},B_{2,3},x_{3},B_{1,3},x_{1},B_{1,2}, \end{array}$$

which forms a cycle of length 6 in the Tanner graph of the BIBD-LDPC code. This sequence satisfies the conditions for a cycle, as each consecutive pair of points and blocks corresponds to a nonzero entry in the incidence matrix, and the sequence is closed. Hence, this point-and-block sequence represents a closed path of length 6 in the Tanner graph, confirming the presence of cycles of length 6 in the BIBD-LDPC code. In the following, we explore methods to systematically identify and count such cycles.

It is evident that the point-and-block sequence described above can be simplified to the point sequence expressed as $x_{1},x_{2},x_{3}$. Without loss of generality, any three distinct points selected from the point set (X) can form a similar point-and-block sequence as in (1). Consequently, there exist numerous cycles of length 6 in the Tanner graphs of BIBD-LDPC codes. This confirms that the girth of BIBD-LDPC codes constructed from BIBD(κ,1;υ) is 6.

### 3.3. A Method for Counting Short Cycles of BIBD-LDPC Codes

Since the girth of BIBD-LDPC codes is 6, we focus on analyzing cycles of length 2i in the Tanner graph of a BIBD-LDPC code for i≥3. Based on the cycle analysis in the preceding subsections, it is clear that the existence of cycles of length 2i is closely tied to the point-and-block sequence described in (1) or, equivalently, the point sequence in (2). These sequences provide a systematic way to identify and characterize cycles in the Tanner graph, enabling a deeper understanding of the cycle structure and its impact on the performance of BIBD-LDPC codes.

According to the theory of quasi-cyclic LDPC (QC-LDPC) codes with a girth of *g*, Equation (4) in [11] provides the necessary and sufficient condition for the existence of cycles of length 2i for g≤2i<2g [35]. Therefore, for the constructed BIBD-LDPC codes, the sequence (2) precisely represents cycles of length 2i for 6≤2i≤10. From (2), it follows that identifying cycles of length 2i is equivalent to finding *i* points in X such that any *t* adjacent points in these *i* points do not lie in the same block for 3≤t≤i. Consequently, determining the number of cycles of length 2i can be formulated as a combinatorial problem. It is important to note that for 2i≥12, the existence of cycles of length 2i depends on the presence of cycles of length *j* for j≤i, such as cycles of length 6. The analysis of these longer cycles involves more complex sequences derived from (2). In the following, we focus on cycles of length 2i in the Tanner graph of BIBD-LDPC codes, which can be divided into three parts:

- Selection of *i* points from X: The number of ways to choose *i* distinct points from the υ points in X is given by the combination formula expressed as
  $$C_{\upsilon }^{i}=\frac{\upsilon !}{i!(\upsilon -i)!}.$$
  This quantity is denoted by $P_{1}$.
- Exclusion of invalid cases: Among the chosen *i* points, some cases must be excluded where any *t* adjacent points (for 3≤t≤i) lie in the same block. These cases require a detailed combinatorial analysis, and the number of such invalid cases is denoted by $P_{2}$.
- Counting valid cycles of length 2i: For the remaining valid selections of *i* points, the number of cycles of length 2i that can be formed is denoted by $P_{3}$. This involves determining how many distinct cycles of length 2i exist for each valid set of *i* points. This is analogous to the circle permutation problem but without considering the order of permutation. A simple counting argument shows that
  $$P_{3}=\frac{(i-1)!}{2}.$$
  This formula accounts for the number of distinct cycles that can be formed from *i* points, treating rotations and reflections as identical.

Based on the above analysis, the total number of cycles of length 2i in the Tanner graph of a BIBD-LDPC code is given by

$$\left(P_{1}-P_{2}\right)\times P_{3}.$$

Since $P_{1}$ and $P_{3}$ can be directly calculated using the corresponding formulas based on the parameters of the BIBD, our focus shifts to determining $P_{2}$, which represents the number of invalid cases where *t* adjacent points (for 3≤t≤i) lie in the same block. Once $P_{2}$ is computed, the number of cycles of length 2i can be determined for specific values of *i*. In the following, we apply the proposed cycle-counting method to calculate the number of cycles of lengths 6 and 8 in the Tanner graph of the BIBD-LDPC code constructed from BIBD(κ,1;υ). It is noted that parameters $i,t,P_{1},P_{2},P_{3},\kappa ,$ and υ are consistently used in the following two subsections to maintain a unified framework for counting cycles of different lengths in the Tanner graphs of BIBD-LDPC codes.

#### 3.3.1. Number of Cycles of Length 6

In this case, we only consider i=3, and parameter *t* takes only one value, namely 3. Therefore, for the cases of $P_{2}$, the only scenario to consider is when three points chosen from X lie in the same block. In other words, we calculate the number of cases where any three points of X are contained within a single block. For any block in B with κ≥3, the number of ways to choose three points from the block is given by

$$C_{\kappa }^{3}=\frac{\kappa (\kappa -1)(\kappa -2)}{3!}.$$

Since the total number of blocks in the BIBD is

$$\frac{\upsilon (\upsilon -1)}{\kappa (\kappa -1)},$$

the total number of invalid cases ($P_{2}$) is

$$P_{2}=\frac{\upsilon (\upsilon -1)(\kappa -2)}{3!}.$$

Additionally, the values of $P_{1}$ and $P_{3}$ are calculated as follows:

$$P_{1}=C_{\upsilon }^{3}=\frac{\upsilon (\upsilon -1)(\upsilon -2)}{3!},$$

and

$$P_{3}=\frac{(3-1)!}{2}=1.$$

Combining $P_{1}$ and $P_{2}$, the exact number of cycles of length 6 ($N_{6}$) is

(3) $$\begin{array}{cc} N_{6} & =\left(P_{1}-P_{2}\right)\times P_{3}\\ \; & =(\frac{\upsilon (\upsilon -1)(\upsilon -2)}{3!}-\frac{\upsilon (\upsilon -1)(\kappa -2)}{3!})\times 1\\ \; & =\frac{\upsilon (\upsilon -1)(\upsilon -\kappa )}{3!}. \end{array}$$

After a simple verification, it can be observed that even for κ=2, the number of cycles of length 6 remains consistent with the expression for $N_{6}$ in (3). Specifically, substituting κ=2 into the formula expressed as

$$N_{6}=\frac{\upsilon (\upsilon -1)(\upsilon -\kappa )}{3!},$$

yields

$$N_{6}=\frac{\upsilon (\upsilon -1)(\upsilon -2)}{3!}.$$

This result aligns with the combinatorial interpretation, as the case of κ=2 inherently prevents the formation of cycles of length 6 due to the inability to have three points in a single block. Thus, the formula remains valid for κ=2, confirming its generality.

#### 3.3.2. Number of Cycles of Length 8

In this case, i=4, and we first assume κ≥4. The *t* parameter takes two values: 3 and 4. Therefore, cases of $P_{2}$ can be partitioned into two distinct categories: (a) all four points in the same block (t=4) and (b) three points in the same block and one point in a different block (t=3).

The first class of cases of $P_{2}$ is that all four points are in the same block (t=4). This corresponds to the scenario where any four points of X lie in the same block. The number of such cases, denoted by $P_{21}$, is calculated as follows:

(1) For each block, the number of ways to choose four points is expressed as
  $$C_{\kappa }^{4}=\frac{\kappa (\kappa -1)(\kappa -2)(\kappa -3)}{4!}.$$
(2) The total number of blocks in the BIBD is expressed as
  $$\frac{\upsilon (\upsilon -1)}{\kappa (\kappa -1)}.$$
(3) Thus, the total number of cases where four points lie in the same block is expressed as
  $$\begin{array}{cc} P_{21} & =\frac{\upsilon (\upsilon -1)}{\kappa (\kappa -1)}\times \frac{\kappa (\kappa -1)(\kappa -2)(\kappa -3)}{4!}\\ \; & =\frac{\upsilon (\upsilon -1)(\kappa -2)(\kappa -3)}{4!}. \end{array}$$

The second class of cases of $P_{2}$ is occurs when three points lie in the same block and one point is in a different block (t=3). This corresponds to the scenario where any three of the four points lie in the same block and the fourth point lies in a different block. The number of such cases, denoted by $P_{22}$, is calculated as follows:

(1) For each block, the number of ways to choose three points is expressed as
  $$C_{\kappa }^{3}=\frac{\kappa (\kappa -1)(\kappa -2)}{3!}.$$
(2) The fourth point must be chosen from the remaining (υ−κ) points (since it cannot be in the same block as the first three points).
(3) The total number of blocks in the BIBD is
  $$\frac{\upsilon (\upsilon -1)}{\kappa (\kappa -1)}.$$
(4) Thus, the total number of cases where three points lie in the same block and the fourth point lies in a different block is expressed as
  $$\begin{array}{cc} P_{22} & =\frac{\upsilon (\upsilon -1)}{\kappa (\kappa -1)}\times \frac{\kappa (\kappa -1)(\kappa -2)}{3!}\times \left(\upsilon -\kappa \right)\\ \; & =\frac{\upsilon (\upsilon -1)(\kappa -2)(\upsilon -\kappa )}{3!}. \end{array}$$

Combining these two cases, the total number of invalid cases ($P_{2}$) is

$$\begin{array}{cc} P_{2} & =P_{21}+P_{22}\\ \; & =\frac{\upsilon (\upsilon -1)(\kappa -2)(\kappa -3)}{24}+\frac{\upsilon (\upsilon -1)(\kappa -2)(\upsilon -\kappa )}{6}. \end{array}$$

Since

$$\begin{array}{cc} P_{1} & =C_{\upsilon }^{4}\\ \; & =\frac{\upsilon (\upsilon -1)(\upsilon -2)(\upsilon -3)}{4!}. \end{array}$$

and

$$\begin{array}{cc} P_{3} & =\frac{(4-1)!}{2}=3, \end{array}$$

the number of cycles of length 8 $\left(N_{8}\right)$ is expressed as

(4) $$\begin{array}{cc} N_{8} & =\left(P_{1}-P_{2}\right)\times P_{3}\\ \; & =\frac{\upsilon (\upsilon -1)}{8}\times \left(\left(\upsilon -2\right)\left(\upsilon -3\right)-\left(\kappa -2\right)\left(4\upsilon -3\kappa -3\right)\right), \end{array}$$

where κ≥4.

When κ=3, it is evident that the cases of $P_{2}$ (where all four points lie in the same block) cannot occur, since each block contains only three points. Therefore, $P_{21}=0$. For κ=3, the number of cycles of length $\left(N_{8}\right)$ is determined solely by the second cases of $P_{2}$, where three points lie in the same block and the fourth point lies in a different block. Substituting κ=3 into the expression for $P_{22}$, we have

$$\begin{array}{cc} P_{22} & =\frac{\upsilon (\upsilon -1)(\kappa -2)(\upsilon -\kappa )}{3!}\\ \; & =\frac{\upsilon (\upsilon -1)(\upsilon -3)}{6}. \end{array}$$

Since $P_{21}=0$, the total number of invalid cases ($P_{2}$) is

$$\begin{array}{cc} P_{2} & =P_{21}+P_{22}\\ \; & =\frac{\upsilon (\upsilon -1)(\upsilon -3)}{6}. \end{array}$$

Hence, the number of cycles of length 8 ($\left(N_{8}\right)$) for κ=3 is

$$\begin{array}{cc} N_{8} & =\left(P_{1}-P_{2}\right)\times P_{3}\\ \; & =\frac{\upsilon (\upsilon -1)(\upsilon -3)(\upsilon -6)}{8}. \end{array}$$

When κ=2, the block size is too small to accommodate the conditions required for two classes of cases of $P_{2}$. Specifically, the first class of cases requires four points to lie in the same block, which is impossible, since κ=2, and the second class of cases requires three points to lie in the same block, which is also impossible, since κ=2. Thus, both $P_{21}$ and $P_{22}$ are zero, and the total number of invalid cases ($P_{2}$) is

$$P_{2}=P_{21}+P_{22}=0+0=0.$$

Hence, the number of cycles of length 8 $\left(N_{8}\right)$ for κ=2 is

$$\begin{array}{cc} N_{8} & =\left(P_{1}-P_{2}\right)\times P_{3}\\ \; & =\frac{\upsilon (\upsilon -1)(\upsilon -2)(\upsilon -3)}{8}. \end{array}$$

Based on the above analysis, it can be observed that for κ=2 and κ=3, the number of cycles of length 8 $\left(N_{8}\right)$ is consistent with the expression for $N_{8}$ derived in (4) with κ≥4. Thus, the general expression for $N_{8}$ in (4) remains valid for κ=2 and κ=3, demonstrating its generality across different block sizes. This confirms that the cycle-counting method is robust and applicable to a wide range of BIBD-LDPC codes.

## 4. Short Cycles of Specific BIBD-LDPC Codes

In this section, we provide specific examples of BIBD-LDPC codes and determine their short-cycle distributions. By applying the cycle-counting method developed in the previous sections, we calculate the number of cycles of lengths 6 and 8 for these codes. This analysis highlights the relationship between the parameters of the BIBD (e.g., κ,υ) and the cycle structure of the resulting LDPC codes.

First, we provide some important parameters of BIBD-LDPC codes. Based on a BIBD(κ,1;υ), we can construct a BIBD-LDPC code of length υυ−1υυ−1κκ−1κκ−1, the parity-check matrix of which has a column weight of κ and a row weight of $\left(\upsilon -1\right)/\left(\kappa -1\right)$. The code rate is about (1−κκ−1κκ−1υ−1(υ−1)). The detailed parameters of the BIBD-LDPC codes are presented in Table 1. Second, we provide some existing BIBDs. For convenience, we do not introduce the construction of the existing BIBDs and only provide their existence conditions. We summarize some existing BIBDs in Theorem 1.

**Theorem** **1.** *A BIBD(κ,1;υ) exists for κ = 3, 4, 5, 6, and 7 if one of the following conditions is satisfied.*

*(1)*  *If υ≡1mod6 or υ≡3mod6, there exists a BIBD(3,1;υ).*
*(2)*  *If υ≡1mod12 or υ≡4mod12, there exists a BIBD(4,1;υ).*
*(3)*  *If υ≡1mod20 or υ≡5mod20, there exists a BIBD(5,1;υ).*
*(4)*  *If υ≡1mod15 or υ≡6mod15 for υ≠ 16, 21, 36, 46, 51, 61, 81, 166, 226, 231, 256, 261, 286, 316, 321, 346, 351, 376, 406, 411, 436, 441, 471, 501, 561, 591, 616, 646, 651, 676, 771, 796, and 801, there exists a BIBD(6,1;υ).*
*(5)*  *If υ=42i+1 for i≠ 1, 2, 3, 5, 6, 12, 14, 17, 19, 22, 27, 33, 37, 39, 42, 47, 59, and 62 or υ=42t+7 for i≠ 3, 19, 34, and 39, there exists a BIBD(7,1;υ).*

Based on the cycle-number equations derived in Section 3.3, we can directly determine the number of short cycles in BIBD-LDPC codes constructed from a BIBD(κ,1;υ). By applying Theorem 1, we can obtain a BIBD and subsequently construct a BIBD-LDPC code. Using this approach, the number of short cycles in the Tanner graphs of the constructed BIBD-LDPC codes can be accurately counted. These results are recorded in Table 2.

Notably, the proposed cycle-counting method achieves O(1) complexity, as the enumeration of cycles of lengths 6 and 8 reduces to closed-form expressions parameterized by υ. This contrasts sharply with existing approaches that require greater computational complexity. For instance, counting cycles with a length of 6 in a BIBD-LDPC code constructed from BIBD(κ,1;υ) using the competitive message-passing algorithm proposed in [10] incurs complexity of O(gυυ−1υυ−1κκ−1κκ−1), whereas our method requires only two subtractions, three multiplications, and one division operation.

## 5. Simulation Results

In the following simulations, we assume an additive white Gaussian noise (AWGN) channel, binary phase-shift keying (BPSK) modulation, and the sum-product algorithm (SPA) with 50 iterations for decoding.

**Figure 1:**
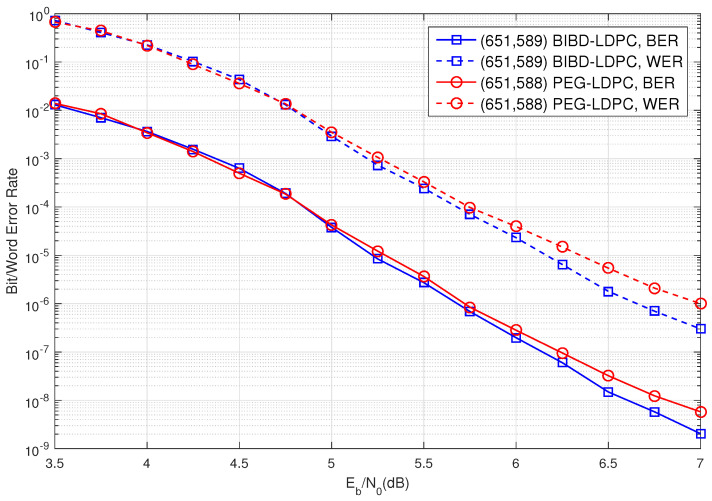
The error performance of the constructed (651,589) BIBD-LDPC code and the comparable (651,588) LDPC code designed by the PEG algorithm in [16].

Consider BIBD(3,1;63). This design consists of 63 points and 651 blocks. Using its incidence matrix, we construct a (3,31)-regular (651,589) BIBD-LDPC code. The bit-error rate (BER) and word-error rate (WER) of this code, decoded using the sum-product algorithm (SPA), are illustrated in Figure 1. For comparison, we also construct a (3,31)-regular (651,588) LDPC code based on the progressive edge-growth (PEG) algorithm [16], referred to as the PEG-LDPC code. The BER/WER performance of this PEG-LDPC code is also included in Figure 1. From Figure 1, it is evident that the (651,589) BIBD-LDPC code outperforms the (651,588) PEG-LDPC code by approximately 0.3 dB at a WER of $10^{-6}$. Additionally, the BIBD-LDPC code exhibits no error floor down to a BER of $2\times 10^{-9}$. Notably, the (3,31)-regular (651,589) BIBD-LDPC code contains 39,060 cycles of length 6 and 1,669,815 cycles of length 8.

**Figure 2:**
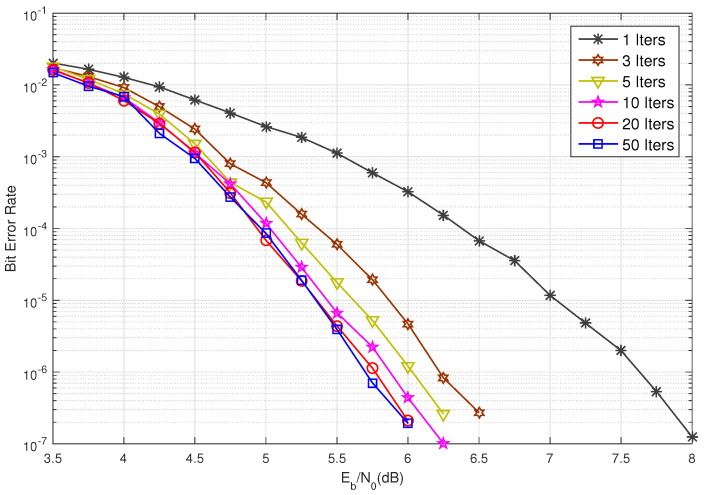
The decoding convergence rate of the constructed (925,851) BIBD-LDPC code.

**Figure 3:**
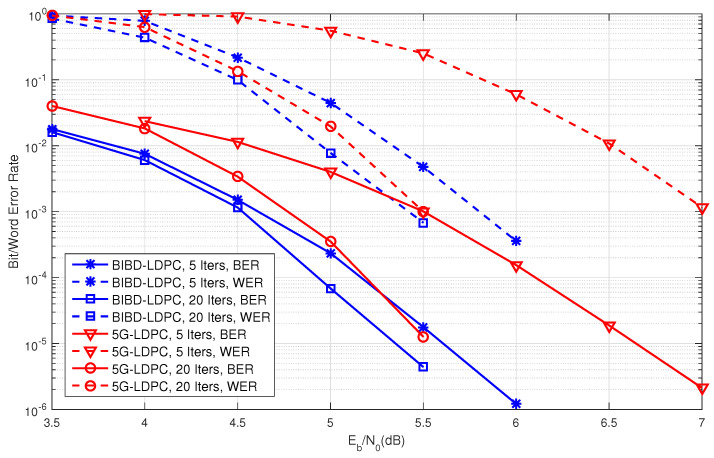
The error performance of the constructed (925,851) BIBD-LDPC code and the comparable (925,851) 5G-LDPC code in [36].

Consider BIBD(3,1;75). This design consists of 75 points and 925 blocks. The null space of the incidence matrix of this BIBD yields a (3,37)-regular (925,851) BIBD-LDPC code. The BER performance of this code under iterative decoding using the sum-product algorithm (SPA) with 1, 3, 5, 10, 20, and 50 iterations is depicted in Figure 2. As shown in Figure 2, the iterative decoding of this code converges very quickly. The BER performance curves for 20 and 50 iterations are nearly identical, indicating convergence. At a BER of $10^{-6}$, the performance gap between 5 and 50 iterations is only 0.25 dB, while the gap between 10 and 50 iterations is approximately 0.1 dB. Furthermore, this BIBD-LDPC code exhibits a low error floor, as demonstrated in Figure 2. For the (3,37)-regular (925,851) BIBD-LDPC code, the numbers of cycles of lengths 6 and 8 are 66,600 and 3,446,550, respectively. For performance benchmarking, we compare the constructed (3,37)-regular (925,851) BIBD-LDPC code against the standard (651,589) 5G-LDPC code specified in [36]. Figure 3 presents the BER/WER performance under SPA decoding with both 5 and 20 iterations. It can be seen from Figure 3 that the constructed BIBD-LDPC code achieves approximately 0.2 dB coding gain at a BER of $10^{-5}$ with 20 decoding iterations compared to the 5G-LDPC code, and the performance advantage becomes more pronounced (about 1 dB gain at BER $=10^{-5}$) when using only 5 iterations. These comparative results validate that the proposed BIBD construction maintains performance with competitive with modern 5G standards while offering faster convergence, a critical advantage for latency-sensitive applications.

**Figure 4:**
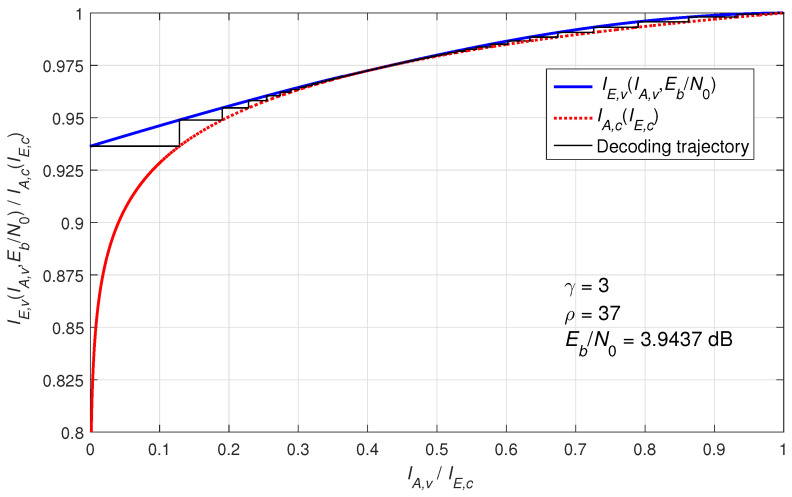
An EXIT chart example for the (3,37)-regular LDPC code ensemble.

To evaluate the performance of our proposed BIBD-LDPC codes, we employ extrinsic information transfer (EXIT) chart analysis to examine their convergence behavior [37]. Figure 4 illustrates a typical EXIT chart for regular LDPC codes operating over a binary-input AWGN channel with parameters of γ=3 and ρ=37. The EXIT chart utilizes extrinsic mutual information as its key metric, comprising two characteristic curves: (1) The upper solid blue curve ($I_{E,V}$ vs. $I_{A,V}$) represents the variable-node processors (VNPs), showing the output extrinsic mutual information ($I_{E,V}$) as a function of input a priori information ($I_{A,V}$). (2) The lower dashed red curve ($I_{A,C}$ vs. $I_{E,C}$) characterizes the check-node processors (CNPs), depicting output extrinsic mutual information ($I_{E,C}$) versus input a priori information ($I_{A,C}$). (Note that this curve is conventionally plotted with reversed axes for EXIT chart representation.) The iterative decoding process manifests as a trajectory bouncing between these curves, where extrinsic information from VNPs becomes a priori information for CNPs and vice-versa. Beginning at the (0,0) point (complete uncertainty), successful decoding is achieved when the trajectory converges to (1,1) (perfect information transfer and error-free decoding). This graphical representation vividly demonstrates the information exchange dynamics between VNPs and CNPs. The widening “tunnel” between the curves at higher SNR values accelerates decoder convergence. The shown SNR (slightly above 3.9437 dB) represents the decoding threshold for the (3,37) code ensemble. Below this critical threshold, the tunnel closes, preventing the trajectory from reaching the (1,1) point and resulting in nonzero error rates.

## 6. Conclusions

In this paper, we proposed a method for constructing LDPC codes based on a BIBD(κ,1;υ). We analyzed the cycle structure of the constructed LDPC codes and determined their girth. Numerical results show that the constructed LDPC codes exhibit excellent performance under iterative decoding. Furthermore, we presented a method for counting cycles in the Tanner graphs of these codes, specifically enumerating cycles of lengths 6 and 8. This method can be extended to systematically count cycles of lengths greater than 8 (e.g., 10, 12, etc.). However, the complexity of the analysis increases significantly with the cycle length, primarily due to the need to account for the presence of existing shorter cycles (such as those of lengths 6 and 8) and their interactions within the Tanner graph.

## Figures and Tables

**Table 1:** Parameters of BIBD-LDPC codes constructed from a BIBD(κ,1;υ).

BIBDs	BIBD-LDPC Codes			
	Code Length	Row Weight	Column Weight	Code Rate
(3,1;υ)	υ(υ−1)/6	(υ−1)/2	3	≈(1−6/(υ−1))
(4,1;υ)	υ(υ−1)/12	(υ−1)/3	4	≈(1−12/(υ−1))
(5,1;υ)	υ(υ−1)/20	(υ−1)/4	5	≈(1−20/(υ−1))
(6,1;υ)	υ(υ−1)/30	(υ−1)/5	6	≈(1−30/(υ−1))
(7,1;υ)	υ(υ−1)/42	(υ−1)/6	7	≈(1−42/(υ−1))

**Table 2:** The number of short cycles of BIBD-LDPC codes constructed from a BIBD(κ,1;υ).

BIBDs	Cycle Lengths and Numbers of BIBD-LDPC Codes		
	Length 4	Length 6	Length 8
(3,1;υ)	0	υ(υ−1)(υ−3)/6	$3C_{\upsilon }^{4}-\upsilon \left(\upsilon -1\right)\left(\upsilon -3\right)/2$
(4,1;υ)	0	υ(υ−1)(υ−4)/6	$3C_{\upsilon }^{4}-\upsilon \left(\upsilon -1\right)\left(4\upsilon -15\right)/4$
(5,1;υ)	0	υ(υ−1)(υ−5)/6	$3C_{\upsilon }^{4}-3\upsilon \left(\upsilon -1\right)\left(2\upsilon -9\right)/4$
(6,1;υ)	0	υ(υ−1)(υ−6)/6	$3C_{\upsilon }^{4}-\upsilon \left(\upsilon -1\right)\left(4\upsilon -21\right)/2$
(7,1;υ)	0	υ(υ−1)(υ−7)/6	$3C_{\upsilon }^{4}-5\upsilon \left(\upsilon -1\right)\left(\upsilon -6\right)/2$

## Data Availability

The data that support the findings of this research are available from the corresponding author upon reasonable request.

## Abbreviations

The following abbreviations are used in this manuscript:

LDPC	Low-density parity check
BIBD	Balanced incomplete block design
BIBD-LDPC codes	LDPC codes constructed from BIBDs
ML	Maximum likelihood
RC	Row–column
QC-LDPC	Quasi-cyclic LDPC
N6	Number of cycles of length 6
N8	Number of cycles of length 8
AWGN	Additive white Gaussian noise
BPSK	Binary phase-shift keying
SPA	Sum-product algorithm
PEG	Progressive edge growth
PEG-LDPC	LDPC code based on the progressive edge-growth algorithm
BER	Bit-error rate
WER	Word-error rate
